# Integrated analysis of clinical and genetic factors on the interindividual variation of warfarin anticoagulation efficacy in clinical practice

**DOI:** 10.1186/s12872-023-03321-9

**Published:** 2023-05-31

**Authors:** Bao Sun, Siqing Ma, Feiyan Xiao, Jianquan Luo, Mouze Liu, Wenhui Liu, Zhiying Luo

**Affiliations:** 1 Department of Pharmacy, The Second Xiangya Hospital, Central South University, No.139, People’s Middle Street, Furong District, Changsha City, 40013 Hunan Porv. China; 2 Institute of Clinical Pharmacy, Central South University, Changsha, China; 3 Department of Pharmacy, Hunan Institute for Tuberculosis Control, Changsha, China; 4 Department of Pharmacy, Hunan Chest Hospital, Changsha, China; 5 Center for Clinical Trial and Research, The Second Xiangya Hospital, Central South University, Changsha, China

**Keywords:** Warfarin, Polymorphism, PTTR, Efficacy, Pharmacogenomics

## Abstract

**Aim:**

The anticoagulation effect of warfarin is usually evaluated by percentage of time in therapeutic range (PTTR), which is negatively correlated with the risk of warfarin adverse reactions. This study aimed to explore the effects of genetic and nongenetic factors on anticoagulation efficacy of warfarin during different therapeutic range.

**Methods:**

We conducted an observational retrospective study aiming at evaluating the impact of clinical and genetic factors on PTTR from initial to more than six months treatment. This analysis included patients with heart valve replace (HVR) surgery who underwent long-term or life-long time treatment with standard-dose warfarin for anticoagulation control in Second Xiangya Hospital. All patients were followed for at least 6 months. We genotyped single nucleotide polymorphisms in *VKORC1* and *CYP2C9* associated with altered warfarin dose requirements and tested their associations with PTTR.

**Results:**

A total of 629 patients with intact clinical data and available genotype data were enrolled in this study, and only 38.63% patients achieved good anticoagulation control (PTTR > 0.6). Clinical factors, including male gender, older age, overweight, AVR surgery and stroke history, were associated with higher PTTR. Patients with *VKORC1* -1639AA genotype had significantly higher PTTR level compared with GA/GG genotype carriers only in the first month of treatment. Patients with CYP2C9*3 allele had higher PTTR compared with CYP2C9*1*1 carriers. Moreover, compared with *VKORC1* -1639 AG/GG carriers, INR > 4 was more likely to be present in patients with AA genotype. The frequency of CYP2C9*1*3 in patients with INR > 4 was significantly higher than these without INR > 4.

**Conclusion:**

We confirmed the relevant factors of warfarin anticoagulation control, including genetic factors (VKORC1 -1639G > A and CYP2C9*3 polymorphisms) and clinical factors (male gender, older age, overweight, AVR surgery and stroke history), which could be helpful to individualize warfarin dosage and improve warfarin anticoagulation control during different treatment period.

**Supplementary Information:**

The online version contains supplementary material available at 10.1186/s12872-023-03321-9.

## Introduction

Despite the arrival of new oral anticoagulants, warfarin remains one of the most commonly prescribed oral anticoagulants for the prevention and treatment of thromboembolic episodes, and it remains the only choice for oral anticoagulant in patients with mechanical heart valve [1, 2]. The clinical use of warfarin is challenging due to its narrow therapeutic index and huge inter-individual variability in warfarin maintain dose requirement [3, 4]. Patients usually have to maintain the international normalized ratio (INR) within the therapeutic range (2.0—3.0) depending on their indications. Genetic variations are considered the major factor that influences the warfarin dose required, especially variants in the genes encoding the main drug metabolic enzymes cytochrome P450 (CYP) 2C9 (*CYP2C9*) and the drug target vitamin K epoxide reductase complex subunit 1 (*VKORC1*) [5].

The quality of warfarin anticoagulation control can be evaluated by PTTR, expressed by the percent of time within the target range [6]. A higher PTTR value is associated with a reduced risk of thromboembolic or hemorrhagic adverse events and indicates a better anticoagulation control [7, 8]. A large part of prospective clinical trials, which were usually designed to test the effect of genotype-based dosing on warfarin anticoagulation control, usually defined PTTR as the primary outcome [9, 10]. The National Institute for Health and Care Excellence recommends PTTR > 65% for optimal anticoagulation control with vitamin K antagonists [11]. One meta-analysis showed that time in therapeutic range (TTR) in earliest 3 months was longer in genotype-based dosing algorithms compared with standard vitamin K antagonist dosing algorithms [12]. Moreover, personalization of warfarin dose based on *CYP2C9* and *VKORC1* genotypes showed a longer TTR compared to traditional strategies [13]. In addition, YP4F2 polymorphisms could not result in any favorable clinical outcomes except for the reduction of supra-therapeutic INR.

Vast variation in PTTR can be also showed among difference populations, the mean PTTR was significantly higher in Australia (82%) compared with Singapore (58%) [14, 15]. PTTR level also had wide inter-patient variability, and multiple factors including age, gender, ethnicity, SAMe-TT_2_R_2_ score, drug combination, other complications, adherence to treatment and genetic factors had previously evidenced to be associated with PTTR individual difference [16, 17]. More recently, Eriksson et al. firstly showed that *ASPH* rs4379440 polymorphism was associated with PTTR during the first 3 months through a Genome Wide Association Study [18]. However, one recent study based on Brazil population failed to testify this association. Among these genetic factors, polymorphisms in the *CYP2C9* and *VKORC1* genes are usually be supposed to be related with PTTR difference, and a number of pharmacogenomics studies have been conducted to investigate the association of *CYP2C9* and *VKORC1* polymorphisms with PTTR inter-individual difference [19, 20]. However, the results between studies did not reach consensus, none of the published studies evaluated the association between genetic/non-genetic factors and PTTR during different time periods of treatment. Hence, the aim of this study was to explore the associationof PTTR with polymorphisms of CYP2C9 and VKORC1 in Chinese population.

## Method

### Study population

This study was conducted in compliance with the Declaration of Helsinki. This project was approved by the Ethics Committee of the Institute of Clinical Pharmacology at Central South University (CTXY-110005) and the trial was previously registered: ChiCTRONC-11001532. Written informed consent was received from participants before enrolment, and each patient had been regularly observed for at least 6 months as follow-up period. Enrolled participants were identified by random numbers.

### Clinical variables collection, follow-up method and genotyping

We respectively followed-up patients who underwent HVR surgery and initial warfarin therapy in the Cardiac Surgery Department of Xiangya Hospital and Second Xiangya Hospital from February 2017 to December 2018. Inclusion criteria were age ≥ 18 years; under HVR surgery and treated with warfarin; being follow-up for at least 6 months. Exclusion criteria were malignant tumor; under the age of 18 years; severe liver or kidney function dysfunction; pregnant and parturient women. Electronic medical records was reviewed for clinical information, including: age, sexual, height, weight, smoking and drinking habit, combined disease (hypertension, diabetes mellitus, coronary heart disease, stroke history, etc.), combined treatment (herbs, aspirin, amiodarone, fluconazole and so on), clinical indications (mechanical HVR, biological HVR, mitral valve replacement (MVR), aortic valve replacement (AVR), and tricuspid valve replacement (TVR)) and INR values of each test. Concomitant medication use was recorded during follow-up and those medications were classified as drugs which could increase or decrease INR level.

**Fig. 1:**
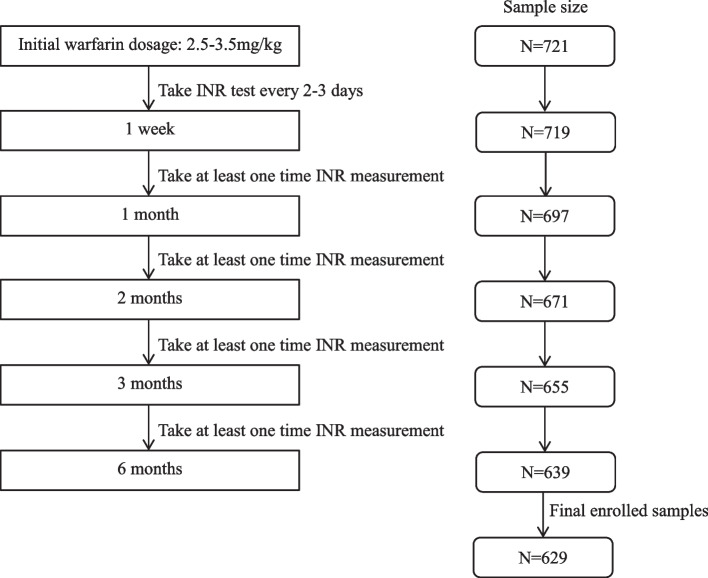
Study flowchart

All patients received an initial dose of 2.5 mg to 3.5 mg warfarin daily. All patients returned regularly to adjust therapeutic dose on the basis of INR result. The patient treatment and followed-up strategy was shown in Fig. 1. We respectively recorded the INR, warfarin dosage and the drug combination status after each follow-up visit.

Peripheral whole blood samples (2 ml) were collected and stored in a -20℃environment. The genomic DNA was extracted using a commercial Geomic DNA Purification Kit (Wizard Genomic DNA Purification Kit, A1620; Promega) according to the protocol. The DNA samples were stored at -20℃ until being used. The polymorphisms of *VKORC1* -1639G > A and CYP2C9*3 in this study were genotyped by pyrosequencing as described in our previously published manuscript [3].

### The determination of INR and PTTR

2 ml of peripheral blood were collected in tube with EDTA as anticoagulant and then centrifuged for 15 min with 2500 rpm. The values of PT and INR are tested with STAGO STAR Evolution fully automatic clotting analyzer. The therapeutic range of INR was in accordance with the American College of Cardiology/American Heart Association (ACC/AHA) guideline for the management of patients with valvular heart disease. Target INR range depended on the position of valve and the presence of atrial fibrillation (AF). The therapeutic INR was between 2.0 and 3.0 for HVR patients, and was between 2.5 and 3.5 for patients with TVR. Rosendaal linear interpolation method was used to calculate warfarin PTTR [6]. The primary outcome of this study was PTTR. The secondary outcomes included the frequency of good anticoagulation control (defined as PTTR ≥ 60%), and the frequency of over-anticoagualtion (patients with INR greater than 4.0) [21, 22].

### Statistical analysis

The sample size was estimated by Power and Sample Size 3.1.2 as described in our previous published manuscript [23]. Means and standard deviations were calculated for continuous variables, and frequencies and percentages were calculated for categorical variables. The association between covariates and outcomes was performed using SPSS Statistic version 19.0 (SPSS, Inc, Chicago, Illinois). A χ^2^ test calculated deviation from Hardy–Weinberg equilibrium. T test (for continuous variables) and χ2 test (for categorical variables) were used in analyzing the difference of characteristics between groups.

## Results

### Characteristics of samples enrolled in this study

A total of 629 patients with intact clinical data and available genotype data were finally enrolled in this study based on the inclusion and exclusion criteria, as shown in Fig. 1. The basic characteristics of patients were presented in Table 1. Most patients (88.39%) were under mechanical HVR (MHVR) surgery and required lifetime warfarin anticoagulation therapy. Only 23 patients had prescribed drugs which might increase INR levels by influencing the pharmacokinetics or pharmacodynamics of warfarin. According to the follow-up strategy, patients had taken 10.9 times INR tests on average and median follow-up time was 291 days. Average PTTR gradually increased with follow-up time, as shown in Supplementary Fig. 1, and PTTR tended to be stable after 2 months of treatment. After 6 months of follow-up, only 38.63% patients achieved good anticoagulation control (PTTR > 0.6). A total of 533 patients got stable warfarin dosage during follow-up time.

**Table 1:** Characteristics of enrolled patients

Variables	Number (total samples = 629)
Sexual, M/F	244 (38.79%) /385 (61.21%)
Age, y	46.71 ± 10.33
Weight, kg	160.41 ± 7.77
High, cm	57.49 ± 9.92
Smoking habit	67 (10.65%)
Drinking history	36 (5.72%)
Combined diseases	
Hypertension	58 (9.22%)
CHD	19 (3.02%)
T2D	13 (2.07%)
AF	268 (42.61%)
Stroke history	28 (4.45%)
Infectious endocarditis	30 (4.77%)
Digestive tract disease	14 (2.23%)
Gallbladder disorders	21 (3.34%)
Hepatitis	17 (2.70%)
Hyperthyroidism	16 (2.54%)
Pausimenia	93 (14.79%)
MHVR	556 (88.39%)
BHVR	73 (11.61%)
MAZE	99 (15.74%)
AF after Surgery	169 (26.87%)
Drugs increase INR	23 (3.66%)
Drugs decrease INR	0
Follow-up time, media (25th-75th IQR), day	291 (225–442)
INR measurements, media (25th-75th IQR)	9 (7–11)
INR > 4	229(36.40%)
INR > 100	19 (3.02%)
PTTR (6 M)	55.88 ± 25.0
PTTR (6 M) > 80%	125 (19.87%)
PTTR (6 M) > 60%	288 (45.79%)
PTTR (6 M) < 40%	183 (29.09%)
*VKORC1* -1639AA	520 (82.67%)
*VKORC1* -1639AG	104 (16.53%)
*VKORC1* -1639GG	5 (0.79%)
*CYP2C9* *1*1	574 (91.26%)
*CYP2C9* *1*3 /*3*3	55 (8.74%)

*CHD* Coronary heart disease, *T2D* Type 2 diabetes, *AF* Atrial fibrillation, *MHVR* Mechanical heart valve replacement, *BHVR* Bioprosthetic heart valve replacement, *MAZE* Wolf Mini-maze surgery

### Influence of clinical factors on PTTR difference

**Fig. 2:**
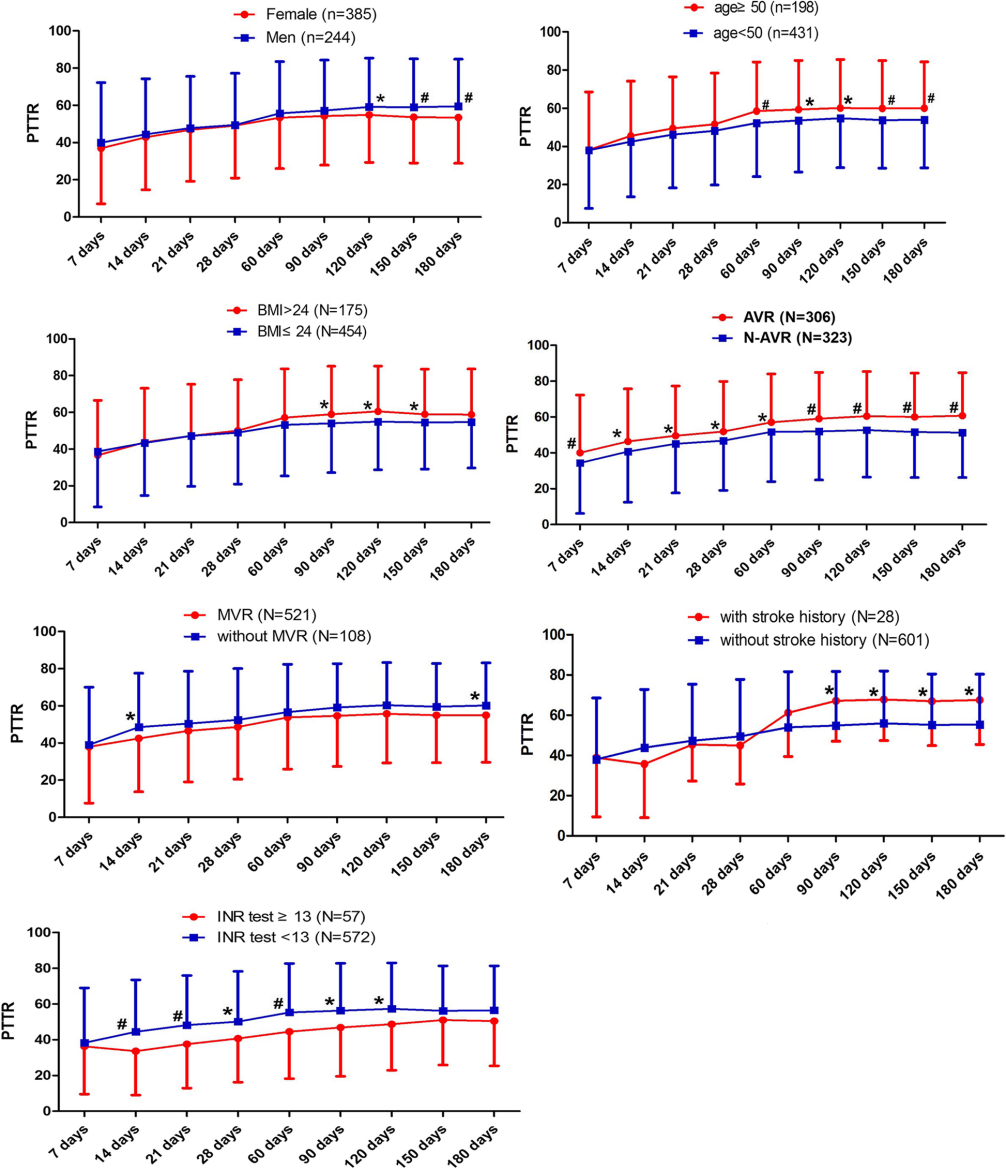
Association between clinical factors and PTTR difference during follow-up period

We firstly evaluated the effect of clinical characteristics on PTTR inter-individual difference, and found thatgender, age, BMI, AVR, combined with stroke history and INR measure times were significantly associated with anticoagulation control, as shown in Supplementary Table 1. In detail, these data showed that male patients, older patients, overweight patients, patients with AVR surgery and patients with stroke history were more likely to have better anticoagulation control, with data shown in Fig. 2. The median INR test time was 9 in 6-month follow-up period (IQ1-IQ3 = 7–11). More frequent INR measurements (> 13 times) was associated with higher PTTR and better anticoagulation efficacy. However, this advantage gradually became insignificant with the extension of treatment time.

### Influence of genetic polymorphisms on PTTR

**Fig. 3:**
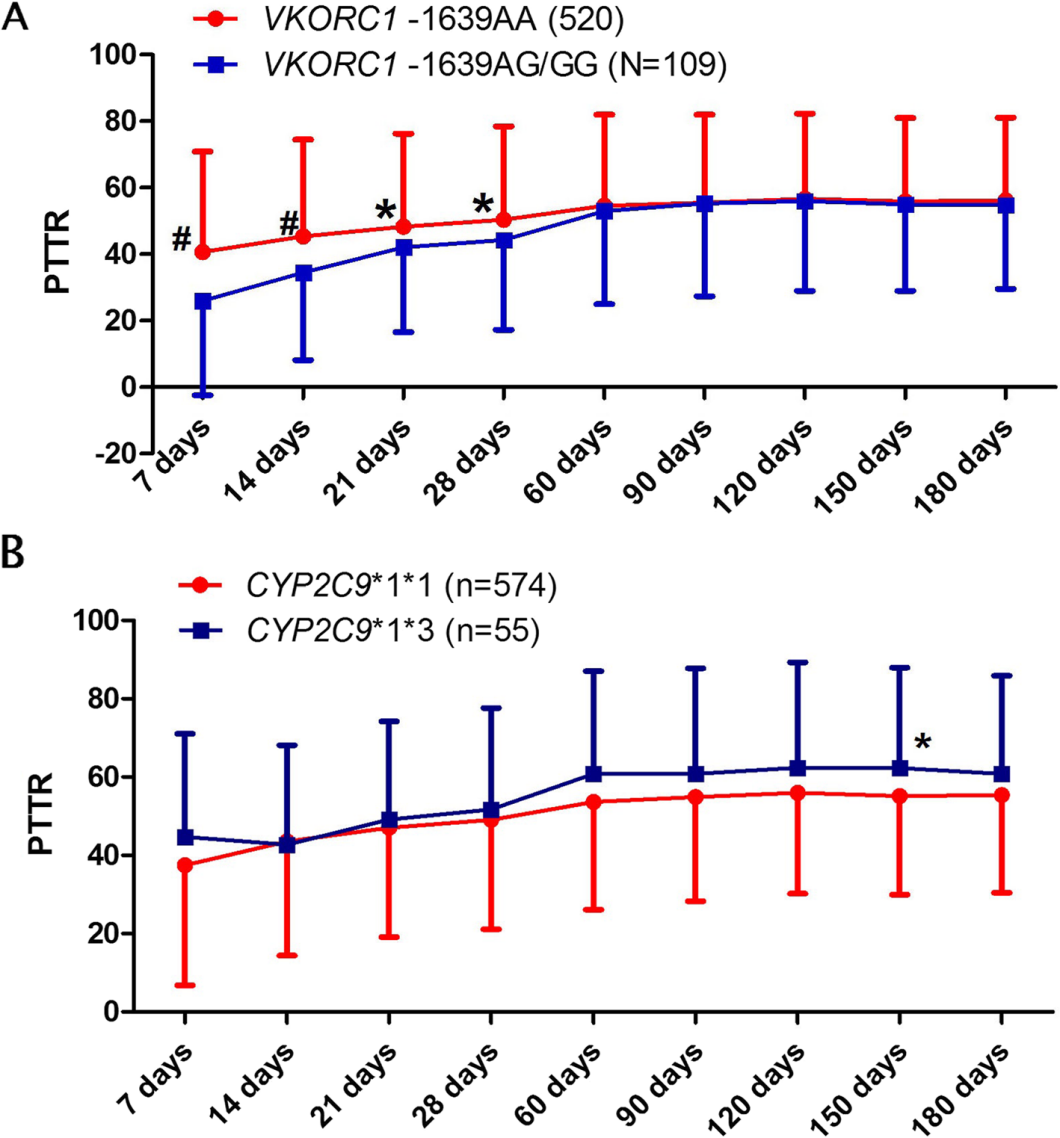
PTTR varies with follow-up time in patients with different genotypes of *VKORC1* -1639G > A and CYP2C9*3. 3A, *VKORC*1 -1639G > A polymorphism and PTTR change; 3B, CYP2C9*3 polymorphism and PTTR change

In this study population, the prevalence of *VKORC1* -1639AA genotype, AG genotype and GG genotype were 82.67%, 16.53% and 0.8%, respectively. The G allele frequency was 9.06%. The genotype frequencies for CYP2C9*1*1, *1*3, *3*3 were 91.25%, 8.75% and 0%, respectively. The allele frequencies of CYP2C9*1*1 and *1*3 were in Hardy–Weinberg equilibrium (*P*-value were 0.94 and 0.23, respectively). Patients with *VKORC1* -1639AA genotype had significantly higher PTTR level compared with GA/GG genotype carriers in the first therapeutic month, as shown in Fig. 3A. However, this difference became inconspicuous as the treatment continued and number of times of warfarin dose adjustment increased. Patients with CYP2C9*1*3 allele had higher PTTR compared with CYP2C9*1*1 carriers, and only significant difference was observed in 5 months PTTR (Fig. 3B, *P* = 0.044).

### Factors associated with good anticoagulation control

**Fig. 4:**
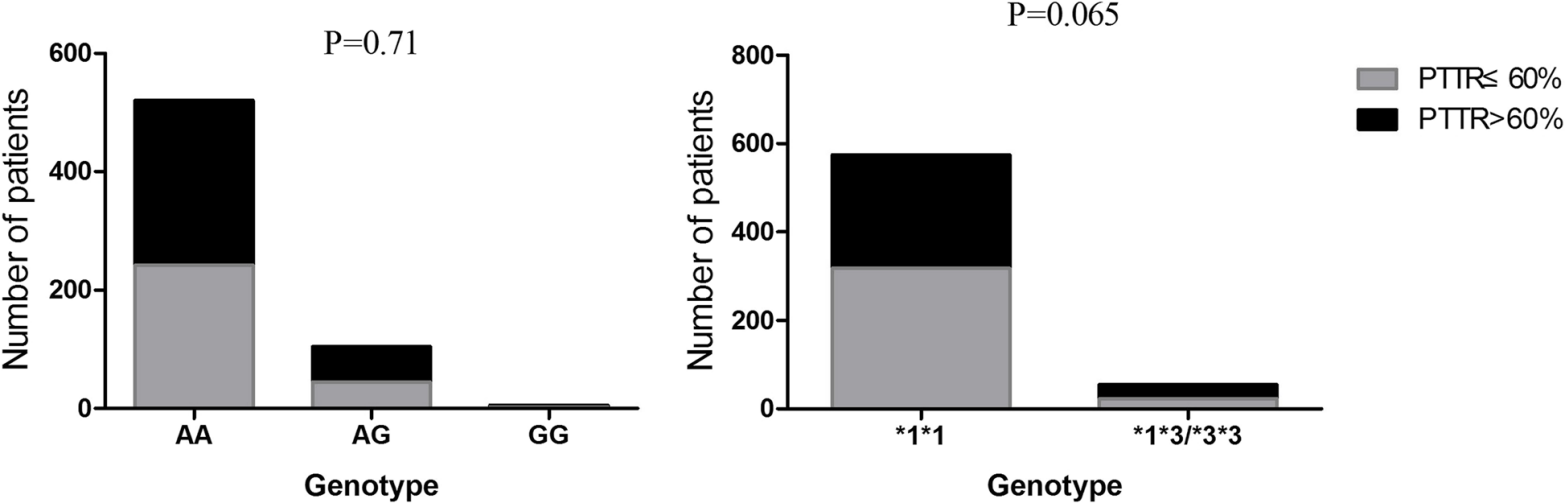
Association of distribution of VKORC1-1639G > A and CYP2C9*3 genotypes with INR > 4

Good anticoagulation control was defined as PTTR > 60% after 6 months of follow-up. Table 2 reported the univariate analysis of the rate of good anticoagulation control. Compared with younger patients, older patients (especially aged more than 50 years) were confirmed to have a significantly higher rate of good anticoagulation control (OR 1.57 (95%CI 1.12–2.21), *P* = 0.01). Males had higher PTTR > 60% rate than females (OR 1.55 (95%CI 1.127–2.14), *P* = 0.009). Patients with PTTR ≥ 60% had higher height and weight than those with PTTR < 60% (*P* = 0.025 and 0.015 respectively). However, the BMI level showed no statistic significant difference between groups. The material and type of valve replacement also showed significant difference between patients with good anticoagulation control and those without good coagulation control, as shown in Table 2. We compared the genotype distributions of *VKORC1* -1639G > A and CYP2C9*3 polymorphismsin good anticoagulation patients and found that the genotype frequencies of *VKORC1* -1639G > A and CYP2C9*3 showed no significant difference between patients with good anticoagulation control and those without anticoagulation control, as shown in Fig. 4 (*P* = 0.71 and *P* = 0.065 respectively).

**Table 2:** Clinical and genetic data of patients with PTTR ≥ 60% and PTTR < 60%

Variables	PTTR ≥ 60% (*N* = 288)	PTTR < 60% (*N* = 341)	P
Age, y	47.94 ± 10.38	45.68 ± 10.20	0.006
Age ≥ 50y	106 (36.81%)	92 (26.98%)	0.01
Age < 50y	182 (63.19%)	249 (73.02%)	
High, cm	161.17 ± 7.73	159.77 ± 7.75	0.025
Weight, kg	58.54 ± 10.19	56.60 ± 9.60	0.015
BMI	22.46 ± 3.17	22.12 ± 3.07	0.16
BMI ≥ 24	53 (47.74%)	58 (52.25%)	0.67
BMI < 24	235 (45.37%)	283 (54.63%)	
Sexual, F/M	160 (55.56%)/128 (44.44%)	225 (65.98%)/116 (34.02%)	0.009
Smoking habit	35 (12.15%)	32 (9.23%)	0.30
Drinking history	21 (7.29%)	15 (4.40%)	0.12
MHVR	247 (44.26%)	311 (55.74%)	0.042
BHVR	41 (57.74%)	30 (42.26%)	
AVR	166 (57.64%)	140 (41.06%)	9.08E-8
MVR	230 (79.86%)	291 (85.34%)	0.072
TVR	19 (6.60%)	14 (4.11%)	0.005
Hypertension	32 (11.11%)	26 (7.62%)	0.16
CHD	13 (4.51%)	6 (1.76%)	0.06
T2D	6 (2.08%)	7 (2.05%)	1.00
AF	133 (46.18%)	135 (39.59%)	0.11
Stroke history	18 (6.25%)	10 (2.93%)	0.052
MAZE	47 (16.32%)	52 (15.25%)	0.74
AF after surgery	86 (29.86%)	83 (24.34%)	0.13
Infectious endocarditis	11 (3.82%)	19 (5.57%)	0.35
Digestive tract disease	9 (3.13%)	5 (1.47%)	0.18
Gallbladder disorders	6 (2.08%)	15 (4.40%)	0.12
Hepatitis	7 (2.43%)	10 (2.93%)	0.81
Hyperthyroidism	8 (2.78%)	8 (2.35%)	0.80
Pausimenia	38 (13.19%)	55 (16.13%)	0.31
INR measurements, media (25th-75th IQR)	7 (7–11)	9 (8–11)	0.25
*VKORC1*			0.30
AA	242 (84.03%)	275 (80.56%)	
AG + GG	46 (15.97%)	66 (19.35%)	
*CYP2C9*			0.065
*1*1	256 (88.89%)	318 (93.26%)	
*1*3	32 (11.11%)	23 (6.74%)	

*BMI* Body mass index, *AVR* Aortic valve replacement, *MVR* Mitral valve replacement, *TVR* Tricuspid valve replacement

### Factors associated with over-anticoagulation frequency

Patientshad INR measurement result > 4 at least for once were defined as patients with over-anticoagulation, which is associated with bleeding events. The analysis results showed that both genetic and nongenetic factors were significantly associated with INR > 4 (Table 3). The results showed that older patients (OR 0.65 (95%CI 0.47–0.90), *P* = 0.01), over-weight patients (OR 0.67 (95%CI 0.45–0.99), *P* = 0.044) were not prone to occur INR > 4 events. Moreover, female patients were more inclined to have INR > 4 compared with male patients (OR 1.55 (95%CI 1.10–2.18), *P* = 0.014). Further analysis showed that patients with type 2 diabetes (T2D) or hyperthyroidism were more likely to have INR > 4 compared with patients without these complications (*P* = 0.018 for T2D and 0.015 for hyperthyroidism, respectively). Moreover, INR > 4 was significantly more prevalent in patients prescribed with concomitant medications (such as aspirin, amiodarone, fluconazole), which might increase INR compared to patients without these drugs (OR 5.49 (95%CI 2.22–13.57), *P* = 2.09E-4). Compared with *VKORC1* -1639 AG or GG carriers, INR > 4 is more likely to occur in patients with AA genotype (OR 1.63 (95%CI 1.03–2.58), *P* = 0.037). The frequency of CYP2C9*1*3 in patients with INR > 4 was significantly higher than those without INR > 4 (OR 2.26 (95%CI 1.29–3.95), *P* = 0.005).

**Table 3:** Difference of clinical and genetic factors between patients with INR > 4 or without INR > 4

Variables	INR ≤ 4 (*N* = 229)	Without INR > 4 (*N* = 400)	P
Age, y	48.57 ± 10.10	45.65 ± 10.33	0.001
Age ≥ 50y	89 (38.86%)	109 (27.25%)	0.003
Age < 50y	140 (61.14%)	291(72.75%)	
High, cm	159.04 ± 7.49	161.20 ± 7.82	0.001
Weight, kg	56.74 ± 10.12	57.92 ± 9.78	0.82
BMI	22.37 ± 3.33	22.21 ± 2.99	0.27
Sexual, F/M	155 (67.68%)/74 (32.32%)	230 (57.50%)/170 (42.50%)	0.014
Pausimenia	47 (30.32%)	93 (40.43%)	0.052
Smoking habit	21 (9.17%)	46 (11.5%)	0.42
Drinking history	10 (4.37%)	26 (6.50%)	0.29
MHVR	203 (88.65%)	353 (88.25%)	1.00
BHVR	26 (11.35%)	47 (11.75%)	
AVR, (*n* = 306)	101 (44.10%)	205 (51.25%)	0.24
MVR, (*n* = 521)	208 (40.00%)	313 (60.00%)	4.15E-5
TVR, (*n* = 33)	17 (51.51%)	16 (48.49%)	0.76
Hypertension	19 (8.30%)	39 (9.95%)	0.57
CHD	5 (2.18%)	14 (3.5%)	0.47
T2D	9 (3.93%)	4 (1.0%)	0.018
AF before surgery	104 (45.41%)	164 (41.0%)	0.31
Stroke history	15 (6.55%)	13 (3.25%)	0.069
MAZE	32 (13.97%)	67 (16.75%)	0.43
AF after surgery	72 (31.44%)	97 (24.25%)	0.061
Infectious endocarditis	9 (3.93%)	30 (7.5%)	0.086
Digestive tract disease	6 (2.62%)	8 (2.0%)	0.59
Gallbladder disorders	8 (3.49%)	13 (3.25%)	1.00
Hepatitis	4 (1.75%)	13 (3.25%)	0.32
Hyperthyroidism	11 (4.80%)	5 (1.25%)	0.015
Drug increase INR	13 (5.24%)	8 (2.25%)	2.09E-4
*VKORC1*			0.037
AA	199 (86.90%)	321 (80.25%)	
AG + GG	30 (13.10%)	79 (19.75%)	
*CYP2C9*			0.005
*1*1	199 (86.90%)	375 (93.75%)	
*1*3	30 (13.1%)	25 (6.25%)	

## Discussion

The present study aimed to evaluate the influence of clinical and genetic factors on interindividual difference of warfarin anticoagulation control in a cohort of Chinese patients under HVR surgery. We further determined the association of those factors with PTTR during different treatment stage. This research showed that VKORC1 -1639G > A mutation, the type of HVR surgery and INR test frequency were main causes of PTTR difference in the early stage of treatment (1^st^ treatment month). As the risk of anticoagulant-related bleeding is not predictable at treatment onset, we can speculate that DNA test could helpful in predicting anticoagulation efficacy and appropriately increase INR test could be useful in maintaining desired treatment effect.

As the treatment continued, the advantage of genetic polymorphism and INR test was gradually weakening, and clinical factors, such as gender, age, BMI and stroke history became the main reasons for PTTR interindividual difference. In addition to genetic factors, age, gender, BMI and concomitant drugs that increased INR were widely evidenced to affect warfarin maintenance dose [24]. The difference in PTTR has been studied by other researches that considered non-genetic factors, including demographic factors, medical factors and psychosocial factors as possible modifiers of PTTR [16, 19]. Our data showed that male patients, elderly patients and over-weight patients were more likely to have better anticoagulation control especially after 3 months treatment.

This was in accordance with recently published meta-analyses that women were associated with lower TTR [25], and other research also found that patients with age ≥ 65 years had higher TTR value compared with patients with < 65 years (60 ± 24%) [26]. Our research found that overweight patients had higher PTTR, however inconsistent results was reported in an India cohort that underweight patients had significant higher rate of good anticoagulation control, although the credibility of this research result was impaired by its small sample size [27]. Intriguingly, it has been established that overweight and moderately obese patients with cardiovascular diseases have a better prognosis than patients with normal BMI, giving rise to what is known as an “obesity paradox”. This paradox is further evidenced by a retrospective investigation that the associated risk of venous thromboembolic event (VTE)/stroke was lower in overweight and obese patients on anticoagulation therapy compared to normal weight [28], and some prior meta-analyses that correlated underweight BMI patients with high risks of VTE and bleeding when using warfarin anticoagulation therapy [29]. Although the obesity paradox has been reported for various diseases, it could still be interpreted by some plausible mechanisms. One reasonable explanation is that underweight patients are usually malnourished and more susceptible to illness because of nutrient and vitamin deficiency. Another probable theory suggests that the gut flora in obese population show significant difference with normal BMI population, which results in variation of drug metabolism and function [30].

Besides, we also found that patients under AVR surgery and with stroke history had much higher PTTR. The target INR was guided according to the recommendation of ACC/AHA, and a target INR of 2.0 to 3.0 was usually used for patients after AVR in this study. We also found that patients who had taken 13 INR tests had higher PTTR compared with those who had less than 13 INR tests. Patients usually are asked to take INR test more frequently if their INR level deviation from therapeutic range. Patients had INR level within therapeutic range for two consecutive times usually were asked to take further INR test for a longer interval. Hence, patients with high risks of poor warfarin anticoagulation control might acquire better anticoagulation efficiency of warfarin by taking INR test more frequently. Data of our research showed that *VKORC1* -1639G > A and CYP2C9*3 polymorphisms had no statistically significant association with long-term warfarin anticoagulation control. However, patients with *VKORC1* -1639GA/GG genotypes had significantly lower PTTR compared with AA carriers in the first month. It had been widely evidenced that both *VKORC1* -1639G > A and CYP2C9*3 polymorphisms were significantly associated with warfarin dose difference in different population [31, 32], and personalizing warfarin dose based on *CYP2C9* and *VKORC1* genotypes might be more beneficial compared to traditional strategies [13]. Previous meta-analysis presented that -1639GA and -1639GG carriers required 52% and 102% higher mean daily warfarin dose than -1639AA carriers [33]. As all patients received the same initial dosage in this study, it was easy to understand that patients with -1639GA or -1639GG genotypes required much longer time to adjust therapeutic dosage. As the therapeutic dose adjusted by INR value in the process of treatment, the difference between genotypes was gradually vanished. The adverse effects of warfarin treatment mainly occurred in the first three month [34, 35]. Hence, we can speculate that patients with *VKORC1* -1639G > A mutation could get better anticoagulation control at the beginning of treatment if they had their warfarin dosage modified based on genotype.

The CYP2C9*3 carriers had higher PTTR compared with CYP2C9*1/*1 genotype, but no statistically significant associations were found during long-term warfarin anticoagulation control in our research. Compared with patients with *1/*1 genotype, *1/*2, *1/*3, *2/*2, *2/*3, and *3/*3 carriers required 19.6%, 33.7%, 36.0%, 56.7%, and 78.1% lower warfarin dosage, respectively [36]. Considering the significant difference between warfarin therapeutic dosage between *1/*1 and homozygous genotype, patients with wild type genotype were more likely to benefit more from warfarin anticoagulation therapy in long-term treatment.

Limitations of the present study should be considered. The first limitation is PTTR level is not a substitute for actual clinical anticoagulation outcomes. Due to the low rate of bleeding and embolic events of warfarin anticoagulation treatment in HVR patients, it may be extremely resource-intensive to obtain adequate samples with long-term follow-ups and occurred bleeding or embolic events. On the other hand, we only examined the effects of two SNPs on PTTR, which was not conducive to the discovery of new genetic variants associated with PTTR differences.

In summary, we conducted a pharmacogenomics study to explore factors associated with warfarin anticoagulation control in HVR patients during different period of treatment. Our data highlighted that *VKORC1* -1639G > A and CYP2C9*3 polymorphisms were associated with anticoagulation control only in the initial stage. These finding have the potential for identifying patients who are more likely to have good anticoagulation control of warfarin during different period of treatment, and may further explain clinical benefit of genotyping in individualized treatment of warfarin.

## Supplementary Information

**Additional file 1: Supplemental Table 1.** Association between clinical characteristics and PTTR inter-individual difference. **Supplemental figure 1.** The distribution of PTTR during 6 months treatment.

## Data Availability

The raw data supporting the conclusion of this article will be made available by Zhiying Luo (lzhy199089@csu.edu.cn), without undue reservation, to any qualified researcher.

## References

[1] Johnson JA, Cavallari LH (2015). Warfarin pharmacogenetics. Trends Cardiovasc Med.

[2] Stevens SM, Woller SC, Baumann Kreuziger L (2021). Executive Summary: Antithrombotic Therapy for VTE Disease: Second Update of the CHEST Guideline and Expert Panel Report. Chest.

[3] Luo Z, Li X, Zhu M (2017). Identification of novel variants associated with warfarin stable dosage by use of a two-stage extreme phenotype strategy. J Thromb Haemost.

[4] Wang M, Zeraatkar D, Obeda M (2021). Drug-drug interactions with warfarin: A systematic review and meta-analysis. Br J Clin Pharmacol.

[5] Shukla A, Jain A, Kahalekar V (2019). Mutations in CYP2C9 and/or VKORC1 haplotype are associated with higher bleeding complications in patients with Budd-Chiari syndrome on warfarin. Hep Intl.

[6] Rosendaal FR, Cannegieter SC, van der Meer FJ (1993). A method to determine the optimal intensity of oral anticoagulant therapy. Thromb Haemost.

[7] Lea JC, Floroff CK, Ingemi AI (2019). Impact of time in therapeutic range after left ventricular assist device placement: a comparison between thrombus and thrombus-free periods. J Thromb Thrombolysis.

[8] Carmo J, Ferreira J, Costa F (2017). Non-vitamin K antagonist oral anticoagulants compared with warfarin at different levels of INR control in atrial fibrillation: A meta-analysis of randomized trials. Int J Cardiol.

[9] Kimmel SE, French B, Kasner SE (2013). A pharmacogenetic versus a clinical algorithm for warfarin dosing. N Engl J Med.

[10] Pirmohamed M, Burnside G, Eriksson N (2013). A randomized trial of genotype-guided dosing of warfarin. N Engl J Med.

[11] Rojo M, Roco AM, Suarez M (2020). Functionally Significant Coumarin-Related Variant Alleles and Time to Therapeutic Range in Chilean Cardiovascular Patients. Clin Appl Thromb Hemost.

[12] Belley-Cote EP, Hanif H, D'Aragon F (2015). Genotype-guided versus standard vitamin K antagonist dosing algorithms in patients initiating anticoagulation. A systematic review and meta-analysis. Thromb Haemostasis..

[13] Sridharan K, Sivaramakrishnan G (2021). A network meta-analysis of CYP2C9, CYP2C9 with VKORC1 and CYP2C9 with VKORC1 and CYP4F2 genotype-based warfarin dosing strategies compared to traditional. J Clin Pharm Ther.

[14] Singer DE, Hellkamp AS, Piccini JP (2013). Impact of global geographic region on time in therapeutic range on warfarin anticoagulant therapy: data from the ROCKET AF clinical trial. J Am Heart Assoc.

[15] Bernaitis N, Ching CK, Teo SC (2017). Factors influencing warfarin control in Australia and Singapore. Thromb Res.

[16] Henderson JB, Iyer P, Coniglio AC (2019). Predictors of Warfarin Time in Therapeutic Range after Continuous-Flow Left Ventricular Assist Device. Pharmacotherapy.

[17] Costa GL, Lamego RM, Colosimo EA (2012). Identifying potential predictors of high-quality oral anticoagulation assessed by time in therapeutic international normalized ratio range: a prospective, long-term, single-center, observational study. Clin Ther.

[18] Eriksson N, Wallentin L, Berglund L (2016). Genetic determinants of warfarin maintenance dose and time in therapeutic treatment range: a RE-LY genomics substudy. Pharmacogenomics.

[19] Praxedes MFS, Martins MAP, Mourão AOM (2020). Non-genetic factors and polymorphisms in genes CYP2C9 and VKORC1: predictive algorithms for TTR in Brazilian patients on warfarin. Eur J Clin Pharmacol.

[20] da Silveira M, Melo LA, Gomes FMF (2019). Polymorphisms of CYP2C9*2, CYP2C9*3 and VKORC1 genes related to time in therapeutic range in patients with atrial fibrillation using warfarin. Appl Clin Genet.

[21] Perreault S, Shahabi P, Côté R (2018). Rationale, design, and preliminary results of the Quebec Warfarin Cohort Study. Clin Cardiol.

[22] Wypasek E, Mazur P, Bochenek M (2016). Factors influencing quality of anticoagulation control and warfarin dosage in patients after aortic valve replacement within the 3 months of follow up. J Physiol Pharmacol.

[23] Li D, Luo ZY, Chen Y (2020). LRP1 and APOA1 Polymorphisms: Impact on Warfarin International Normalized Ratio-Related Phenotypes. J Cardiovasc Pharmacol.

[24] Wadelius M, Chen LY, Lindh JD (2009). The largest prospective warfarin-treated cohort supports genetic forecasting. Blood.

[25] Costa Viana C, da Silva Praxedes MF, Freitas Nunes de Sousa WJ (2021). Sex-influence on the time in therapeutic range (TTR) during oral anticoagulation with coumarin derivatives: Systematic review and meta-analysis. Br J Clin Pharmacol..

[26] Marcatto LR, Sacilotto L, Darrieux FC (2016). Age is associated with time in therapeutic range for warfarin therapy in patients with atrial fibrillation. Oncotarget.

[27] Anand A, Kumar R, Gupta A, et al. Development of an interview-based warfarin nomogram predicting the time spent in the therapeutic INR range: A cost-effective, and non-invasive strategy building from a cross sectional study in a low resource setting. Indian Heart J. 2022;74(3):245-8. 10.1016/j.ihj.2022.03.008 PMC9243612 35346664

[28] Zhou Y, Ma J, Zhu W (2020). Efficacy and Safety of Direct Oral Anticoagulants Versus Warfarin in Patients with Atrial Fibrillation Across BMI Categories: A Systematic Review and Meta-Analysis. Am J Cardiovasc Drugs.

[29] Almas T, Muhammad F, Siddiqui L (2012). Safety and efficacy of direct oral anticoagulants in comparison with warfarin across different BMI ranges: A systematic review and meta-analysis. Ann Med Surg.

[30] Gomes AC, Hoffmann C, Mota JF (2018). The human gut microbiota: Metabolism and perspective in obesity. Gut microbes.

[31] Jorgensen AL, FitzGerald RJ, Oyee J (2012). Influence of CYP2C9 and VKORC1 on patient response to warfarin: a systematic review and meta-analysis. PLoS ONE.

[32] Takeuchi M, Kobayashi T, Biss T (2020). CYP2C9, VKORC1, and CYP4F2 polymorphisms and pediatric warfarin maintenance dose: a systematic review and meta-analysis. Pharmacogenomics J.

[33] Yang L, Ge W, Yu F (2010). Impact of VKORC1 gene polymorphism on interindividual and interethnic warfarin dosage requirement–a systematic review and meta analysis. Thromb Res.

[34] Linkins LA, Choi PT, Douketis JD (2003). Clinical impact of bleeding in patients taking oral anticoagulant therapy for venous thromboembolism: a meta-analysis. Ann Intern Med.

[35] Hylek EM, Evans-Molina C, Shea C (2007). Major hemorrhage and tolerability of warfarin in the first year of therapy among elderly patients with atrial fibrillation. Circulation.

[36] Lindh JD, Holm L, Andersson ML (2009). Influence of CYP2C9 genotype on warfarin dose requirements–a systematic review and meta-analysis. Eur J Clin Pharmacol.

