# Supplementary material for: Integrated analysis of clinical and genetic factors on the interindividual variation of warfarin anticoagulation efficacy in clinical practice
Source: BMC Cardiovasc Disord. 2023 May 31;23:279. doi: 10.1186/s12872-023-03321-9 (PMC10230781; doi:10.1186/s12872-023-03321-9)
Supplement: Supplementary file 1 — Additional file 1: Supplemental Table 1. Association between clinical characteristics and PTTR inter-individual difference. Supplemental figure 1. The distribution of PTTR during 6 months treatment. [file 12872_2023_3321_MOESM1_ESM.docx]

Supplemental Table 1. Association between clinical characteristics and PTTR inter-individual difference.

|  | 1W | 2W | 3W | 4W | 2M | 3M | 4M | 5M | 6M |
| --- | --- | --- | --- | --- | --- | --- | --- | --- | --- |
| Sexual (F/M) | 0.24 | 0.50 | 0.67 | 0.89 | 0.32 | 0.18 | 0.045 | 0.008 | 0.004 |
| Pausimenia (yes or no) | 0.232 | 0.185 | 0.132 | 0.161 | 0.09 | 0.143 | 0.097 | 0.287 | 0.583 |
| Age (pearson)  >50y vs. ≤50y | 0.58  0.95 | 0.52  0.24 | 0.53  0.17 | 0.46  0.16 | 0.009  0.009 | 0.015  0.012 | 0.016  0.018 | 0.01  0.004 | 0.005  0.004 |
| BMI  BMI≥24 vs. BMI<24 | 0.47 | 0.93 | 0.97 | 0.70 | 0.11 | 0.038 | 0.015 | 0.048 | 0.075 |
| Smoking habit (yes or no) | 0.19 | 0.66 | 0.53 | 0.86 | 0.92 | 0.84 | 0.63 | 0.40 | 0.26 |
| Drinking habit (yes or no) | 0.17 | 0.75 | 0.95 | 0.42 | 0.58 | 0.45 | 0.45 | 0.33 | 0.16 |
| MHVR (yes or no) | 0.32 | 0.66 | 0.42 | 0.47 | 0.82 | 0.98 | 0.41 | 0.34 | 0.28 |
| MVR (yes or no) | 0.73 | 0.044 | 0.18 | 0.19 | 0.32 | 0.086 | 0.061 | 0.081 | 0.047 |
| AVR (yes or no) | 0.002 | 0.013 | 0.042 | 0.024 | 0.014 | 0.001 | 1.58E-4 | 2.47E-5 | 1.84E-6 |
| TVP (yes or no) | 0.55 | 0.24 | 0.29 | 0.35 | 0.45 | 0.22 | 0.37 | 0.41 | 0.16 |
| TVR (yes or no) | 0.97 | 0.48 | 0.55 | 0.66 | 0.33 | 0.84 | 0.65 | 0.49 | 0.61 |
| DVR (yes or no) | 0.52 | 0.24 | 0.29 | 0.35 | 0.45 | 0.22 | 0.37 | 0.42 | 0.16 |
| Hypertension (yes or no) | 0.67 | 0.20 | 0.34 | 0.49 | 0.35 | 0.64 | 0.66 | 0.45 | 0.30 |
| CHD (yes or no) | 0.91 | 0.85 | 0.54 | 0.51 | 0.20 | 0.074 | 0.11 | 0.066 | 0.096 |
| T2D (yes or no) | 0.22 | 0.24 | 0.048 | 0.46 | 0.61 | 0.52 | 0.43 | 0.36 | 0.41 |
| Stroke history (yes or no) | 0.89 | 0.14 | 0.59 | 0.25 | 0.099 | 0.004 | 0.018 | 0.016 | 0.011 |
| AF after surgery (yes or no) | 0.020 | 0.10 | 0.076 | 0.11 | 0.084 | 0.614 | 0.10 | 0.066 | 0.087 |
| Infectious endocarditis (yes or no) | 0.40 | 0.57 | 0.45 | 0.54 | 0.21 | 0.16 | 0.29 | 0.21 | 0.16 |
| Digestive tract disease (yes or no) | 0.90 | 0.18 | 0.63 | 0.79 | 0.43 | 0.41 | 0.34 | 0.19 | 0.13 |
| Gallbladder disorders (yes or no) | 0.5 | 0.27 | 0.53 | 0.53 | 0.92 | 0.79 | 0.39 | 0.29 | 0.26 |
| hepatitis | 0.37 | 0.65 | 0.97 | 0.61 | 0.67 | 0.46 | 0.67 | 0.57 | 0.58 |
| hyperthyroidism (yes or no) |  |  |  |  |  |  |  |  |  |
| INR measurements (>13 vs. ≤13) | 0.63 | 0.007 | 0.006 | 0.008 | 0.005 | 0.012 | 0.018 | 0.14 | 0.089 |
| VKORC1 AA vs. AG/GA | 3.52E-6 | 1.56E-4 | 0.025 | 0.038 | 0.57 | 0.94 | 0.81 | 0.74 | 0.60 |
| CYP2C9 *1*1 vs. *1*3 | 0.06 | 0.84 | 0.60 | 0.50 | 0.064 | 0.12 | 0.074 | 0.044 | 0.13 |


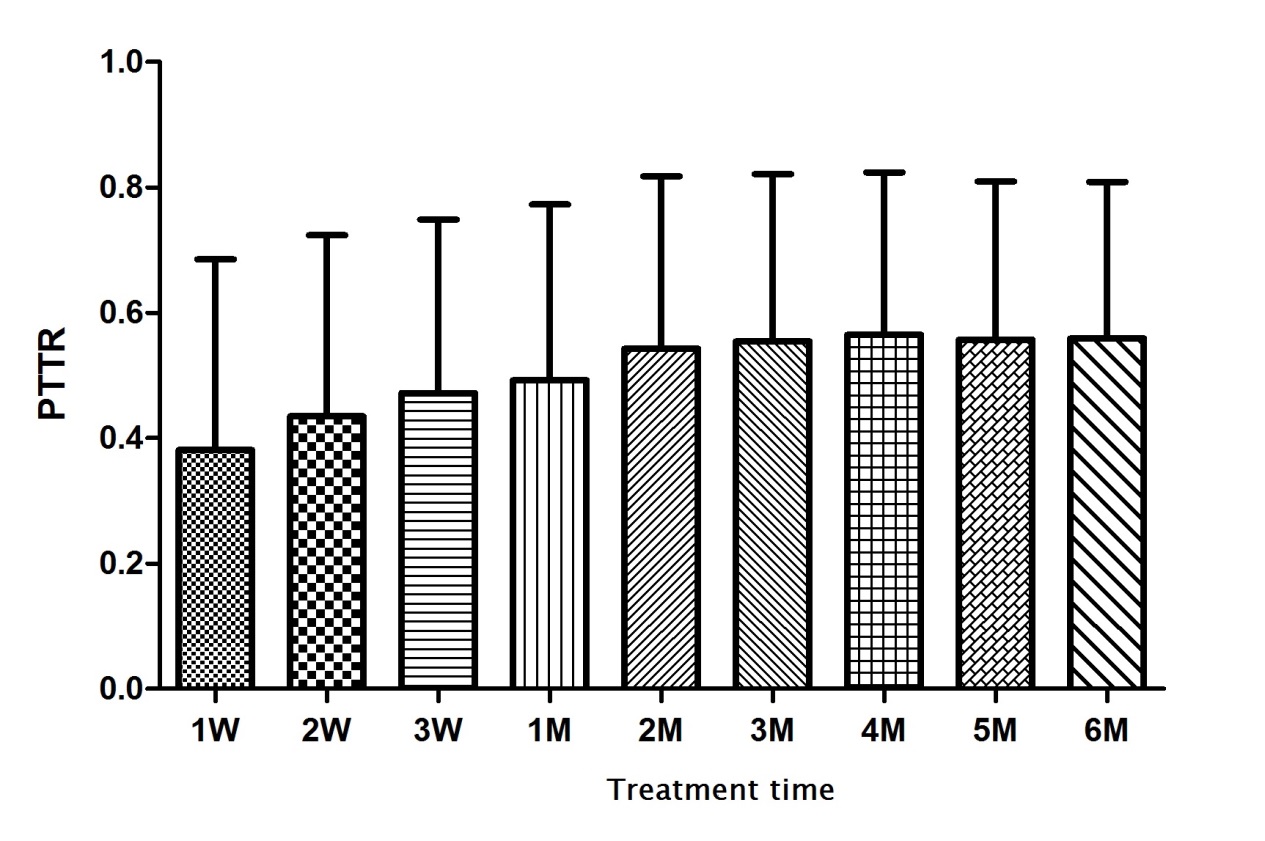


Supplemental figure1. The distribution of PTTR during 6 months treatment.
